# Severity, etiology and outcomes of ionized hypocalcemia in azotemic cats and dogs

**DOI:** 10.3389/fvets.2026.1847148

**Published:** 2026-06-16

**Authors:** Helen S. Philp, Steven E. Epstein, Kate Hopper

**Affiliations:** Department of Veterinary Surgical and Radiological Sciences, UC Davis Weill School of Veterinary Medicine, University of California, Davis, Davis, CA, United States

**Keywords:** acute kidney injury, azotemia, calcium, chronic kidney disease, phosphorus, urinary obstruction

## Abstract

**Objective:**

To describe the severity, timing, etiology, and outcome of ionized hypocalcemia in a population of dogs and cats with all-cause azotemia.

**Methods:**

Medical records of dogs and cats with concurrent ionized hypocalcemia and azotemia, in which ionized calcium (iCa) was measured within 24 h of serum biochemistry testing were retrospectively reviewed from a single institution over a 10-year period (January 1st, 2014, to December 31st, 2023). Ionized hypocalcemia was defined as iCa < 1.21 mmol/L in cats and < 1.30 mmol/L in dogs. Moderate hypocalcemia was defined as iCa < 1.00 mmol/L and severe hypocalcemia as iCa < 0.75 mmol/L. Azotemia was defined as a creatinine concentration ≥ 141 μmol/L (1.7 mg/dL). The primary cause of azotemia was determined by review of the medical record, and diagnostic criteria for acute kidney injury and chronic kidney disease were based on International Renal Interest Society guidelines. Data extracted included signalment, clinicopathologic variables, etiology of azotemia, and outcome. Outcome was defined as survival to discharge or non-survival.

**Results:**

302 cats and 646 dogs met the inclusion criteria. Ionized hypocalcemia was mild in 229/302 (75.8%) cats and 551/646 (85.3%) dogs, moderate in 69/302 (22.8%) cats and 83/646 (12.8%) dogs, and severe in 4/302 (1.3%) cats and 12/646 (1.9%) dogs. The lowest iCa occurred within 48 h of admission in 155/223 (69.5%) cats and 338/465 (72.7%) dogs hospitalized for at least 48 h. In cats, both renal (176/302, 58.3%) and postrenal (126/302, 41.7%) causes of azotemia were common, whereas renal causes predominated in dogs (602/646, 93.2%). Inflammatory/ischemic etiologies were the most common cause of acute kidney injury in both cats (54/90, 60.0%) and dogs (230/383, 60.1%). Ionized calcium showed weak correlations with blood phosphorus (*r* = −0.36 cats, −0.31 dogs), pH (*r* = 0.21 cats, 0.15 dogs), and creatinine concentrations (*r* = −0.30 cats, −0.26 dogs) (all *p* < 0.001). Clinical signs potentially attributable to hypocalcemia were reported in 9/16 (56.3%) severely hypocalcemic patients.

**Conclusion:**

Ionized hypocalcemia in azotemic cats and dogs is generally mild and occurs early in hospitalization. It is only weakly associated with higher blood phosphorus and creatinine concentrations and lower blood pH. Routine monitoring of iCa in azotemic patients is recommended. These findings may not be generalizable to all azotemic patients, as iCa measurement was performed at the discretion of the attending clinician.

## 1 Introduction

Hypocalcemia has been associated with numerous adverse effects including hypotension, systolic dysfunction, arrhythmias, neurologic impairment, new-onset acute kidney injury (AKI) and overall increased mortality in both human and small animal medicine (1–6). Measurement of ionized calcium (iCa) rather than total calcium is recommended because it represents the biologically active fraction. Ionized hypocalcemia is frequently observed in association with azotemia in both cats and dogs (7) and has been reported in 10–26% of cats with chronic kidney disease (CKD) (8), 10–56% of dogs with CKD (9–11), and 34–75% of cats with urethral obstruction (12–14).

In critically ill people with AKI, ionized hypocalcemia has been independently associated with all-cause mortality (15, 16). This effect may be modified with treatment, suggesting a potential opportunity for intervention. In small animals, ionized hypocalcemia has been linked with increased mortality in dogs with AKI (17, 18) but this finding was not replicated in cats (19).

Proposed mechanisms for hypocalcemia in azotemia include hyperphosphatemia, decreased 1,25-dihydroxycholecalciferol formation, and parathyroid hormone resistance due to loss of functional nephrons and/or decreased glomerular filtration rate (20, 21). Hypocalcemia in AKI is thought more likely to be symptomatic because the degree of hyperphosphatemia is often greater than that observed in CKD (22, 23). However, the relationship between serum calcium and phosphate levels in people with AKI is inconsistent (24). The relationships between the severity of ionized hypocalcemia, underlying etiology of azotemia, and concurrent hyperphosphatemia in cats and dogs remain poorly defined, as large-scale comparisons across all causes of azotemia are lacking.

The aims of this study were to describe the severity, timing, and associated etiologies of ionized hypocalcemia, and to evaluate its relationship with serum phosphorus concentration and clinical outcome in a large population of dogs and cats with all-cause azotemia.

## 2 Materials and methods

A computerized search for biochemistry panels^1^ from dogs and cats performed between January 1st, 2014, and December 31st, 2023 was performed using the medical records database at the University of California Davis, William R. Pritchard Veterinary Medical Teaching Hospital. Point of care blood gas and electrolyte panels^2^ that included iCa were identified over the same time period. Animals that had both a biochemistry panel and a blood gas and electrolyte panel were included. Animals without azotemia defined by a blood creatinine concentration ≥141 μmol/L (1.7 mg/dL) on biochemistry with an iCa measured within 24 h of each other were excluded. The medical records for all cases with at least one instance of concurrent ionized hypocalcemia and azotemia were reviewed. If concurrent azotemia and ionized hypocalcemia were documented in the same patient on multiple visits, only the visit with the lowest iCa was included. Animals could only be included once, and all other visits were excluded to avoid duplicate inclusion bias. For hospitalized patients (≥48 h) in which multiple blood gas panels were collected, both the iCa on presentation and the lowest iCa during hospitalization were recorded alongside the timing of the most profound ionized hypocalcemia. Patients with documented azotemia and ionized hypocalcemia only on samples taken more than 24 h apart were excluded. Cases with incomplete medical records sufficient to determine the cause of azotemia and outcome were excluded.

### 2.1 Population data

Data extracted from the electronic medical record included signalment (species, age, sex, reproductive status, bodyweight, breed), history and physical examination findings at presentation, biochemistry, blood gas analysis, ultrasonographic findings, treatments, and outcome. Outcome was defined as survival to discharge or non-survival. Non-survivors were either euthanized or died in hospital.

### 2.2 Etiology of azotemia

The primary cause of azotemia was determined by review of the medical record including history and diagnostic results. When 2 or more potential causes of azotemia were applicable to one patient, the disease process deemed most likely to be the cause following extensive medical record review was selected. Diagnostic criteria for AKI and CKD were based on the International Renal Interest Society (IRIS) guidelines (25). Acute on chronic kidney injury or CKD were diagnosed based on prior clinicopathologic data where available and ultrasonographic evidence consistent with CKD (decreased renal volume, irregular contour, and/or decreased corticomedullary distinction) (26). The etiology of AKI and acute on chronic kidney injury was classified as described in a previous veterinary study (27). Categories included inflammatory/ischemic, infectious, nephrotoxic, neoplastic, other, and unknown. The inflammatory/ischemic category included any cause of severe systemic inflammation or presumed ischemic renal injury. Nephrotoxicosis was defined on the basis of recent ingestion of grapes/raisins (dogs), toxic lilies (cats), or overdose of non-steroidal anti-inflammatory drugs. Pyelonephritis was diagnosed based on a positive urine culture, urine sediment findings, and ultrasonographic findings consistent with pyelonephritis (28). Leptospirosis was diagnosed in cases with a fourfold or higher increase in Leptospira agglutination titer at a single laboratory between acute- and convalescent-phase serum specimens (29). Neoplasia was determined as the etiology when kidney injury was caused by direct renal infiltration of neoplasia (i.e., lymphoma, carcinoma) or resulted from hypercalcemia which was confirmed to be paraneoplastic. “Other” etiology included AKI or acute on chronic injury secondary to non-neoplastic hypercalcemia and glomerulopathies, diagnosed based on the presence of renal proteinuria (urine protein to creatinine ratio > 2) with or without renal biopsy (30).

### 2.3 Clinicopathological parameters

Blood gas and electrolyte analyses were conducted using a point-of-care analyzer (ABL 815, Radiometer Medical A/S, Copenhagen, Denmark) on heparinized blood samples. Institutional reference intervals for this blood gas analyzer were previously generated from 40 healthy cats (31) and 40 healthy dogs (unpublished data). The reference interval for pH was 7.266–7.422 in cats and 7.345–7.433 in dogs. The reference interval for iCa was 1.21–1.45 mmol/L in cats and 1.30–1.46 mmol/L in dogs. Therefore, ionized hypocalcemia was defined using the lower limit of these institution-specific analyzer-derived reference intervals as iCa < 1.21 mmol/L in cats and < 1.30 mmol/L in dogs. Moderate hypocalcemia was defined as iCa < 1.00 mmol/L in both dogs and cats based on previously published veterinary literature evaluating ionized hypocalcemia (7). Severe hypocalcemia was defined as iCa < 0.75 mmol/L in both dogs and cats based on the suggested treatment threshold in the IRIS consensus guidelines for AKI (32). Any reported clinical signs attributed to hypocalcemia in severely hypocalcemic patients were recorded. Serum creatinine and phosphate concentrations were recorded from a serum biochemistry profile through a reference laboratory.

### 2.4 Statistical methods

Statistics were performed using commercially available software.^3^ Numerical data were assessed for normality visually and with the Shapiro–Wilk test. Continuous parametric data were described as mean (± standard deviation) and non-parametric data as median (25th-75th percentile). Spearman rank correlations were calculated to assess the relationship between blood iCa and pH, serum phosphate and creatinine concentrations. A *p* value <0.05 was considered significant. Correlation was considered negligible if *r* = 0.0–0.10, weak if *r* = 0.11–0.39, moderate if *r* = 0.40–0.69, strong if *r* = 0.70–0.89 and very strong if *r* = 0.90–1.00 (33).

## 3 Results

### 3.1 Signalment

**Figure 1:**
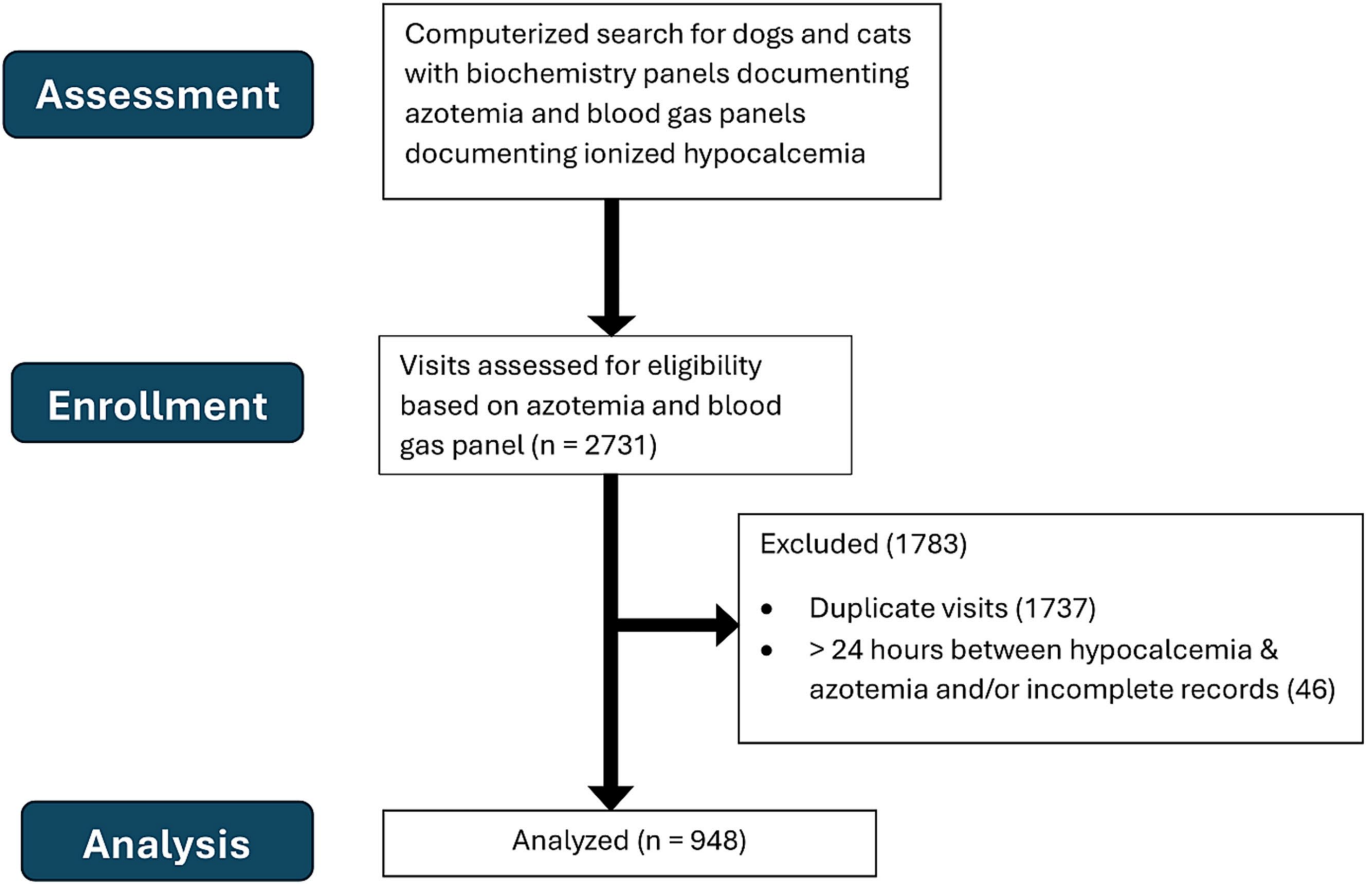
Flowchart to show selection of participants for a study describing ionized hypocalcemia in 948 cats and dogs with all-cause azotemia.

Of 2,731 visits assessed for eligibility, 1737 duplicate visits were excluded. An additional 44 visits were excluded because serum creatinine and iCa measurements were obtained more than 24 h apart, and 2 visits were excluded because of incomplete medical records. Of the remaining 994 visits (321 cats, 673 dogs) identified with azotemia and ionized hypocalcemia during the same visit, 302 cats and 646 dogs satisfied the inclusion criteria (Figure 1). Age was not recorded in 5 cats. The median age of the remaining 297 cats was 9.1 (5.5–12.5) years. In 1 cat, bodyweight was not recorded. In the remaining 301 cats, the median bodyweight was 4.5 (3.6–5.7) kg. Amongst the 302 cats, there were 120 (39.7%) females (5 intact) and 180 (59.6%) males (4 intact). In 2 cats, sex was not recorded. The most common breeds were domestic shorthair (202/302, 66.9%), domestic longhair (31/302, 10.3%), and domestic medium hair (26/302, 8.6%). In 4 dogs, age was not recorded. The median age of the remaining 642 dogs was 9.5 (6.0–12.6) years. In 5 dogs, bodyweight was not recorded. In the remaining 641 dogs, median bodyweight was 12.8 (6.2–29) kg. Amongst the 646 dogs, there were 316 (48.9%) females (51 intact) and 330/646 (51.1%) males (64 intact). The most common breeds were crossbreed (161/646, 24.9%), Labrador retriever (56/646, 8.7%), and chihuahua (22/646, 3.4%).

### 3.2 Etiology of azotemia

Inflammatory/ischemic causes included hypovolemic shock, pancreatitis, peritonitis, pyometra, severe gastroenteritis, pneumonia, diabetic ketoacidosis, trauma, heatstroke, anesthesia-related events, prolonged hypotension, hemorrhagic shock, cardiopulmonary arrest, systolic dysfunction, severe bradyarrhythmias, and congestive heart failure. Common inflammatory/ischemic conditions in cats included cardiogenic/thromboembolic disease (19 cases), hypovolemic shock/trauma (10 cases), diabetic ketoacidosis/hyperosmolar hyperglycemic syndrome (9 cases), pancreatitis (8 cases), septic shock/sepsis (6 cases), and gastrointestinal foreign body or perforation (5 cases). Common inflammatory/ischemic conditions in dogs included sepsis/septic shock/septic peritonitis/pyometra/bile peritonitis (45 cases), cardiogenic disease including congestive heart failure/cardiomyopathy/arrhythmias (31 cases), pancreatitis/diabetic ketoacidosis/hyperosmolar hyperglycemic syndrome (30 cases), hypovolemic or hemorrhagic shock/trauma/hypovolemia (28 cases), gastrointestinal foreign body/obstruction/perforation/gastric dilatation-volvulus (18 cases), and heat stroke (4 cases). Several patients had overlapping inflammatory or ischemic disease processes, and the reported categories reflect the predominant condition identified on medical record review. Infectious causes included pyelonephritis and leptospirosis. Postrenal causes of azotemia included ureteral obstruction, urethral obstruction and urinary tract trauma leading to uroabdomen. In cats, renal causes of azotemia were diagnosed in 176/302 (58.3%) of cats while postrenal causes were responsible for azotemia in 126/302 (41.7%) of cats (Table 1). Amongst the renal causes, AKI was diagnosed in 90/302 (29.8%) of cats. In cats with AKI, an inflammatory/ischemic cause was most common and was documented in 54/90 (60.0%) of cats. Acute on chronic kidney injury was diagnosed in 62/302 (20.5%) of which the majority (33/62, 53.2%) were of unknown cause. Chronic kidney disease was diagnosed as the cause of azotemia in 24/302 (7.9%) of cats. Amongst the 126 cats with postrenal causes of azotemia, 82/126 (65.1%) were diagnosed with ureteral obstruction and 41/126 (32.5%) with urethral obstruction. Three cases (1.0%) were diagnosed with urinary tract trauma. In dogs, renal causes of azotemia were diagnosed in 602/646 (93.2%) and postrenal causes were diagnosed in 44/646 (6.8%) of dogs. Acute kidney injury was diagnosed in 383/646 (59.3%) dogs and acute on chronic kidney injury in 169/646 (26.2%) of dogs. The most common etiologies of AKI were inflammatory/ischemic (230/383, 60.1%) and unknown (72/383, 18.8%). Acute on chronic kidney injury was diagnosed in 169/646 (26.2%) dogs with the majority of cases being of unknown etiology (95/169, 56.2%). Chronic kidney disease as the cause of azotemia was diagnosed in 50/646 (7.7%) dogs. In dogs with postrenal causes, the majority were due to urethral (18, 2.8%) or ureteral (14, 2.2%) obstruction, but trauma (10, 1.5%) and other causes including nephrolithiasis (1, 0.2%) and perineal hernia (1, 0.2%) were also identified.

**Table 1:** Primary etiologies of azotemia in 302 cats and 646 dogs with ionized hypocalcemia and all-cause azotemia.

Etiology	Cats, *n* (%)	Dogs, *n* (%)
Postrenal	126 (41.7)	44 (6.8)
Ureteral obstruction	82 (27.2)	14 (2.2)
Urethral obstruction	41 (13.6)	18 (2.8)
Urinary tract trauma	3 (1.0)	10 (1.5)
Nephrolithiasis	0 (0)	1 (0.2)
Perineal hernia	0 (0)	1 (0.2)
Acute kidney injury	90 (29.8)	383 (59.3)
Inflammatory/ischemic	54 (17.9)	230 (35.6)
Unknown	19 (6.3)	72 (11.1)
Neoplasia	7 (2.3)	5 (0.8)
Nephrotoxic	6 (2.0)	14 (2.2)
Infectious	4 (1.3)	46 (7.1)
Other^a^	0 (0)	16 (2.5)
Acute-on-chronic kidney injury	62 (20.5)	169 (26.2)
Unknown	33 (10.9)	95 (14.7)
Inflammatory/ischemic	16 (5.3)	38 (5.9)
Infectious	10 (3.3)	16 (2.5)
Other^a^	2 (0.7)	19 (2.9)
Neoplasia	1 (0.3)	1 (0.2)
Chronic kidney disease	24 (7.9)	50 (7.7)
Total	302	646

a Other includes 2 cats and 35 dogs with glomerulopathy.

### 3.3 Timing and severity of ionized hypocalcemia

**Figure 2:**
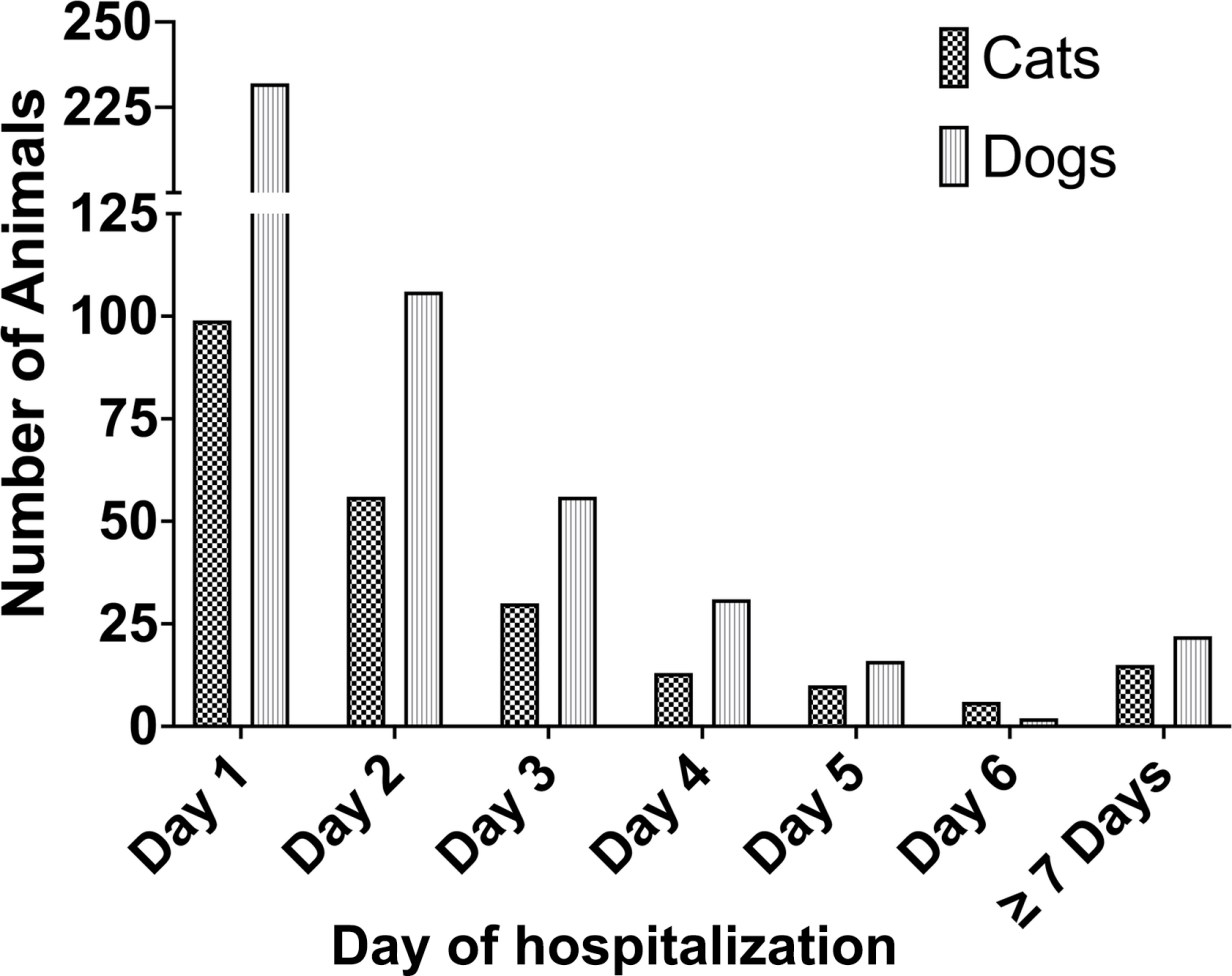
Day of hospitalization in which the lowest documented ionized calcium occurred in 223 azotemic cats and 465 azotemic dogs hospitalized for at least 48 h. iCa, ionized calcium.

On presentation, the median iCa amongst the 302 cats was 1.20 (1.12–1.28) mmol/L. The median lowest iCa concentration was 1.13 (1.02–1.19) mmol/L. At the time of the most profound hypocalcemia documented for all patients, 229 of 302 cats (75.8%) were mildly hypocalcemic, 69 (22.8%) were moderately hypocalcemic, and 4 cats (1.3%) were severely hypocalcemic. The majority of cats (223/302, 73.5%) were hospitalized for at least 48 h. Amongst this population, the lowest iCa was documented within the first 24 h of hospitalization in 99/223 (44.4%) of cats with the lowest iCa documented on Day 2 in 56/223 (25.1%) and Day 3 in 30/223 (13.5%) (Figure 2). The latest documented lowest iCa in 223 cats hospitalized for at least 2 days was on Day 21 in one cat.

On presentation, the median iCa amongst all 646 dogs was 1.22 (1.14–1.29) mmol/L. The median lowest documented iCa concentration for all dogs was 1.14 (1.04–1.21) mmol/L. At the time of the most profound hypocalcemia, 551/646 dogs (85.3%) were mildly hypocalcemic, 83 (12.8%) were moderately hypocalcemic, and 12 dogs (1.9%) were severely hypocalcemic. Of the 646 dogs, 465 (71.8%) were hospitalized for 48 h or more. Amongst this population, the most profound ionized hypocalcemia occurred within the first 24 h of hospitalization in 232/465 dogs (50.0%) with the lowest iCa documented on Day 2 in 106/465 (22.8%) and on Day 3 in 56/465 (12.0%). Clinical signs were reported in 9/16 severely hypocalcemic animals, including altered mentation (*n* = 7) and neuromuscular signs such as tremors or seizures (*n* = 6); several animals exhibited both (Table 2).

**Table 2:** Clinical signs and concurrent diagnoses in severely ionized hypocalcemic cats and dogs with azotemia.

Species	Lowest iCa (mmol/L)	Concurrent diagnoses	Clinical signs recorded
Feline	0.59	Urethral obstruction; severe azotemia; hyperphosphatemia; hyperkalemia	Stuporous; improved after unblocking
Feline	0.61	Acute-on-chronic kidney injury; severe azotemia; hyperphosphatemia	Obtunded
Feline	0.68	Ureteral obstruction; hyperkalemia; atrial standstill	Obtunded
Feline	0.69	Ureteral obstruction; congestive heart failure	Neurologic signs/twitching
Canine	0.57	Dialysis-dependent chronic kidney disease	Seizures
Canine	0.62	Acute kidney injury secondary to pyelonephritis	Pelvic limb twitching
Canine	0.69	Acute kidney injury; myocarditis; systemic hypertension	Obtunded
Canine	0.74	Immune-mediated glomerulonephritis; lymphoma	None documented
Canine	0.48	Heat stroke; hepatic failure; acute kidney injury	Facial twitching
Canine	0.54	Pyometra; duodenal perforation; severe shock	Periarrest/hypotension
Canine	0.65	Acute kidney injury secondary to pyelonephritis	Muscle fasciculations
Canine	0.69	Acute-on-chronic kidney injury; congestive heart failure	None documented
Canine	0.72	Linear foreign body; acute kidney injury	None documented
Canine	0.55	Ruptured pyometra; shock	Stuporous
Canine	0.73	Acute-on-chronic kidney injury	Seizures
Canine	0.74	Acute kidney injury requiring hemodialysis; pancreatitis	None documented

### 3.4 Serum phosphate concentration

On the biochemistry panel measured within 24 h of the lowest iCa, the majority of cats (211/302, 69.9%) were hyperphosphatemic with 78/302 (25.8%) normophosphatemic and 13/302 (4.3%) hypophosphatemic. Amongst the dogs, the contemporaneous biochemistry panel identified that 536/646 (83.0%) were hyperphosphatemic, 102/646 (15.8%) were normophosphatemic and 8/646 (1.2%) were hypophosphatemic at the time of the most profound ionized hypocalcemia.

### 3.5 Correlation between ionized hypocalcemia, pH, phosphorus and creatinine concentration

In cats, there was a weak positive relationship between iCa and blood pH, (*r* = 0.21, *p* = 0.0002), a weak negative correlation between iCa and phosphorus (*r* = −0.36, *p* < 0.0001), and a weak negative correlation between iCa and creatinine (*r* = −0.30, *p* < 0.0001). In dogs, there was a weak positive relationship between iCa and blood pH (*r* = 0.15, *p* = 0.0002), a weak negative relationship between iCa and phosphorus (*r* = −0.31, *p* < 0.0001), and a weak negative correlation between iCa and creatinine (*r* = −0.26, *p* < 0.0001).

### 3.6 Outcome

Amongst the overall population, 204/302 (67.5%) cats were discharged from the hospital while 15/302 (5.0%) died and 83/302 (27.5%) were euthanized. The majority of dogs (406/646, 62.8%) were discharged from the hospital while 40/646 (6.2%) died and 200/646 (31.0%) were euthanized. Among cats with mild, moderate, and severe ionized hypocalcemia, survival to discharge occurred in 155/229 (67.7%), 47/69 (68.1%), and 2/4 (50.0%), respectively. Among dogs, survival to discharge occurred in 350/551 (63.5%), 51/83 (61.4%), and 5/12 (41.7%), respectively. Due to the small number of severely hypocalcemic patients, formal statistical comparisons between severity groups were not performed.

## 4 Discussion

In a large group of azotemic cats and dogs with at least one documented episode of ionized hypocalcemia, the majority of cases were mild, with the most profound hypocalcemia typically occurring within the first 48 h of hospitalization in those admitted for two or more days.

In people, hypocalcemia has been documented in 35–43% of patients with AKI (16, 34) and in 22–30% of those with CKD (35–37). Ionized hypocalcemia has been documented in 10–56% of dogs with CKD (9–11), 10–26% of cats with CKD^8^, and 34–75% of cats with urethral obstruction (12–14). Published cohorts suggest that ionized hypocalcemia is frequent in both dogs and cats with AKI but rarely report a prevalence (7, 18). Unfortunately, it was not possible to determine the prevalence of hypocalcemia in this study of all-cause azotemia given that not all animals had an iCa measured within 24 h of a serum biochemistry panel.

Acute kidney injury was the most common category of azotemia in dogs while postrenal azotemia was more common than AKI in cats in this study. Inflammatory/ischemic causes of AKI were the most frequently identified etiology for AKI in dogs, documented in 60% of this population. These findings are similar to those of a previous study of AKI in dogs reporting inflammatory/ischemic causes in 58% of cases (27). The category of inflammatory/ischemic causes includes a broad range of coexisting conditions which likely accounts for it being so frequent. In cats with postrenal azotemia, ureteral obstruction was more common than urethral obstruction. This may reflect the greater severity and duration of azotemia commonly associated with ureteral obstruction, potentially contributing to the development of hypocalcemia. The markedly higher proportion of postrenal azotemia overall in cats compared with dogs in this study may reflect both true species differences and referral population bias. Ureteral and urethral obstruction are common causes of severe azotemia in cats, and ionized hypocalcemia has previously been reported in 34–75% of cats with urethral obstruction (12–14). In contrast, postrenal azotemia is comparatively less frequent in dogs. In addition, the high caseload of ureteral obstruction managed at our tertiary referral institution likely contributed to the large proportion of cats with postrenal disease. In hospitalized patients, the lowest iCa was documented within the first 24 h of presentation in 50.0% of dogs and 44.4% of cats. In the remaining patients, the lowest documented iCa tended to occur within the first few days of hospitalization with the majority being within the first 48 h. This may be because the severity of azotemia is likely to be greater on initial hospitalization and in many patients, it improves with therapy with a concurrent improvement in ionized hypocalcemia. However, these findings reflect the timing of the lowest documented iCa during hospitalization and were likely influenced by differences in hospitalization duration and frequency of serial monitoring between patients. It is important to note that a significant proportion of both cats and dogs developed ionized hypocalcemia later during hospitalization. Although these animals were azotemic, other contributing factors such as concurrent disease (for example sepsis or pancreatitis), intravenous fluid diuresis, citrated blood product transfusion, and medications including loop diuretics and sodium bicarbonate likely contributed to the development of hypocalcemia (7, 20, 38, 39). These findings highlight the importance of ongoing monitoring of iCa during hospitalization for treatment of azotemia.

In both azotemic cats and dogs, iCa demonstrated statistically significant but weak correlations with blood pH, phosphorus, and creatinine concentrations. In both species the strongest association was with phosphorus (*r* = −0.36 in cats; *r* = −0.31 in dogs); yet this remained weak. Thus, while lower iCa concentrations tended to occur alongside lower blood pH and higher phosphorus and creatinine concentrations, none of these parameters alone appear clinically useful for predicting the degree of ionized hypocalcemia in azotemic cats or dogs. Traditionally, hypocalcemia in azotemia has been attributed to hyperphosphatemia, either through a mass-law effect or reduced calcitriol production, but our findings suggest that these mechanisms alone are insufficient to explain the observed changes. Additional contributors to hypocalcemia in this population may include renal resistance to parathyroid hormone, impaired calcitriol synthesis independent of phosphate, complexing of calcium by uremic toxins, and the effects of systemic inflammation or critical illness on calcium homeostasis. Further studies are needed to explore the specific causes of hypocalcemia and its relationship with hyperphosphatemia in cats and dogs with azotemia. Until then, direct measurement of iCa should be considered in all azotemic cats and dogs, regardless of acid–base status or the degree of hyperphosphatemia or azotemia.

The treatment of hypocalcemia in azotemic patients is often complicated by concerns for increasing the calcium-phosphorus product and promoting metastatic calcification. For this reason, the optimum level at which to initiate therapy is uncertain. Recent IRIS consensus guidelines advocate treatment of ionized hypocalcemia in patients with AKI in the presence of compatible clinical signs and/or severe ionized hypocalcemia (<0.75 mmol/L), and irrespective of hyperphosphatemia (32). The recommended goal of treatment is to eliminate clinical signs rather than normalization of hypocalcemia. Severe hypocalcemia appeared relatively uncommon in this group of azotemic patients and was documented in 4 cats and 12 dogs. Amongst these patients, many had clinical signs that could be due to hypocalcemia. However, each patient had one or more comorbidities, and clinical signs cannot be solely attributed to hypocalcemia. Nonetheless, close monitoring is recommended, and calcium supplementation should be considered if compatible signs develop.

Ionized hypocalcemia has been associated with adverse outcomes in cats and dogs in various disease states (1, 5). However, there are few studies specifically evaluating the association of ionized hypocalcemia and outcome in animals with azotemia. One small study found an association between ionized hypocalcemia and non-survival in cats with urethral obstruction, although there was significant overlap in iCa between survivors and non-survivors (14). Ionized hypocalcemia has been associated with increased mortality in dogs with AKI (17, 18) but did not appear to be prognostic in a study of cats with AKI (19). Ionized hypocalcemia in azotemic and critically ill people has been linked with increased mortality and this relationship appears to be more pronounced when hypocalcemia is sustained over time rather than transient (15, 16). Some evidence has shown that correction of hypocalcemia in azotemic people improves survival (40). However, randomized trials are lacking and some sources even suggest harm in correcting hypocalcemia in certain patient populations (41). Unfortunately, small group sizes in this study precluded meaningful statistical analysis of outcome related to the severity of hypocalcemia as the majority of hypocalcemia was mild. However, 67.5% of cats and 62.8% of dogs were discharged from the hospital.

This study has several limitations, many of which are inherent to its retrospective design. The total number of cases likely underestimates the true prevalence of ionized hypocalcemia, as blood gas analysis and serum chemistry were performed at the discretion of the primary clinician. Many cases were excluded due to the lack of contemporaneous values. In addition, these blood tests are likely more frequently conducted in animals perceived to be more severely ill so the results of this study may not reflect a general population of azotemic animals. Additionally, recurrence of ionized hypocalcemia was likely undocumented in many patients due to inconsistent monitoring and varied duration of hospitalization. Prospective studies are needed to generate more objective data on the incidence, recurrence, clinical impact, and outcomes of hypocalcemia. Such research would also help to clarify the optimal timing, duration, and potential adverse effects of treatment.

## 5 Conclusion

In conclusion, ionized hypocalcemia in a population of azotemic cats and dogs that had evaluation of iCa within 24 h of a biochemistry panel was generally mild and typically occurred within the first 48 h of hospitalization. There was only a weak correlation between lower iCa and lower blood pH and higher phosphorus and creatinine concentrations, suggesting other mechanisms of hypocalcemia are important in this patient population. Further studies are needed to determine the optimal management of ionized hypocalcemia in azotemic patients, but routine monitoring of iCa is recommended.

## Data Availability

The raw data supporting the conclusions of this article will be made available by the authors, without undue reservation.

## Footnotes

1 Chemistry analyzer, Hitachi 917, Roche Diagnostics, Indianapolis, IN.

2 ABL 815, Radiometer Medical A/S, Copenhagen, Denmark.

3 Graph Pad Prism v 10.5, La Jolla, CA.
